# Amplification of glutathione-mediated oxidative stress by catalase in an aqueous solution at hyperthermal temperatures

**DOI:** 10.3164/jcbn.16-28

**Published:** 2017-02-24

**Authors:** Megumi Ueno, Emiko Sekine-Suzuki, Minako Nyui, Ikuo Nakanishi, Ken-ichiro Matsumoto

**Affiliations:** 1 Quantitative RedOx Sensing Team, Department of Basic Medical Sciences for Radiation Damages, National Institute of Radiological Sciences, National Institutes for Quantum and Radiological Science and Technology, 4-9-1 Anagawa, Inage-ku, Chiba-shi, Chiba 263-8555, Japan

**Keywords:** superoxide, hydroperoxy radical, hydrogen peroxide, hyperthermia, redox

## Abstract

The glutathione (GSH)-mediated superoxide (O_2_^•−^) generation in an aqueous solution and relation of hydrogen peroxide (H_2_O_2_) and effect of catalase were investigated. GSH-induced O_2_^•−^ generation in hyperthermal temperatures was measured by the nitroblue tetrazolium (NBT) mehod. Heating an aqueous solution containing GSH caused superoxide from dissolved O_2_. H_2_O_2_ was generated simultaneously in this reaction mixture probably from the hydroperoxy radical (HO_2_^•^), which is equilibrated with O_2_^•−^ in an aqueous condition, and then H_2_O_2_ consumed O_2_^•−^. Coexisting catalase in the reaction mixture, as a result, could increase O_2_^•−^ generation. The catalase-exaggerated extracellular O_2_^•−^ generation could give a harmful effect to living cells. This GSH-induced oxidative stress can be a part of mechanisms of hyperthermia therapy.

## Introduction

Reduced form glutathione (GSH) has been recognized as a major endogenous antioxidant in living cells. GSH is a tripeptide, consisted of glutamic acid, cystein, and glycine. GSH has a thiol on the cysteine moiety and can work as a hydrogen donor, i.e., a direct reductant. Another important biological antioxidative role of GSH is as a coenzyme of glutathione peroxidases (GSH-Px), which catalyzes decomposition of hydrogen peroxide (H_2_O_2_) to harmless water (H_2_O) using GSH, forming oxidized glutathione (GSSG). The reaction catalyzed by glutathione reductase recovers GSH from GSSG using nicotinamide adenine dinucleotide phosphate (NADPH) as a coenzyme. Therefore, it can be stated generally that the GSH protects living cells from oxidative injury directly and/or indirectly.

On the other hand, there are several reports about oxidative action of glutathione and other thiol compounds. In late 1950s, formation of H_2_O_2_ in solution of glutathione with oxygen was reported.^(1)^ Lehninger and Schneider^(2)^ reported that GSH and cysteine cause mitochondrial swelling and that redox state between cytochrome *c* and oxygen is as a determinant. The participation of thiol groups in the oxidative destruction of hemoglobin was examined and be concern.^(3)^ Hoffsten *et al.*^(4)^ reported that the GSH-induced mitochondrial swelling is closely associated with lipid peroxidation. The glutathione induced mitochondrial swelling and lipid peroxidation model has been further studied over subsequent several years.^(5,6)^ In addition, it was reported that the autoxidation of GSH and/or other thiols yields superoxide.^(7)^ Oxidative actions of GSH and other thiols has been reported not so much but occasionally and continuously.^(8–12)^

Recently, temperature dependent generation of superoxide (O_2_^•−^) in an aqueous solution containing GSH was reported.^(13,14)^ Incubating a reaction mixture containing 4-hydroxyl-2,2,6,6-tetramethylpiperidine-*N*-oxyl (TEMPOL) and GSH at 44°C, the TEMPOL shows a steep reduction following a short time delay. This characteristic TEMPOL reduction was halted by adding superoxide dismutase (SOD) to the reaction mixture or bubbling N_2_ gas through the reaction mixture. This result suggests that generation of O_2_^•−^ from dissolved oxygen is related to the observed TEMPOL reduction. Importantly, the temperature dependent reaction of GSH suggests variable behavior of GSH depending on the experimental conditions.

A number of researchers may have experiences that the GSH added as an antioxidant sometimes shows unanticipated paradoxical actions in their test tubes, whether they have reported or ostracized their unexpected results in their experiments. The temperature dependent oxidative actions of GSH through O_2_^•−^ formation may be one reason of the paradoxical biochemical behaviors of GSH. The O_2_^•−^ formation can inevitably generate H_2_O_2_. However, a clear chemical model mechanism of aggressive oxidative action of GSH has not been proposed.

In this paper, GSH-induced O_2_^•−^ generation in hyperthermal temperatures was again confirmed by the nitroblue tetrazolium (NBT) mehod. The NBT method is a conventional assay for determining O_2_^•−^, which can reduce NBT to make a blue formazan having absorption at 560 nm. A relation of H_2_O_2_ simultaneously generated in the reaction mixture and effect of catalase was also investigated. Chemical mechanisms and possible harmful effect of the GSH-induced oxidative effect were described.

## Methods

### Chemicals

GSH, NBT, and catalase from bovine liver (CAT) were purchased from Wako Chemical (Tokyo, Japan). TEMPOL and SOD from human erythrocytes were purchased from Sigma-Aldrich (St. Louis, MO). Other chemicals used in this study were of analytical grade. As the basic solvent of reaction mixtures, 100 mM phosphate buffer (PB) (pH 7.0) containing 0.05 mM diethylenetriaminepentaacetic acid (DTPA) (100 mM PB) was prepared and used for all experiments. Deionized water (Milli-Q system, Merck Millipore, Billerica, MA) was used for preparing 100 mM PB.

### Preparation of reaction mixture and measurement of absorbance

Reaction mixtures containing NBT, GSH, TEMPOL SOD, CAT, and/or H_2_O_2_ were prepared with 100 mM PB to make the final concentrations of contents shown in Table 1. The reaction mixture was kept in a quartz cuvette (1 cm), and was incubated at an arbitrary steady temperature in the spectral photometer (Hitachi, Japan), which was equipped with a water flow temperature control system. Time course of absorbance at 560 nm of the reaction mixture was measured. The measurement was triplicated for each composition of the reaction mixtures.

### X-band electron paramagnetic resonance measurement

The reaction mixture was kept in a vial, and was incubated at 44°C in the water bath. An aliquot (120–130 µl) of the reaction mixture was sampled in a quartz flat cell. Then, the flat cell containing sample solution was set in a TE-mode cavity using a special cell holder and measured as soon as possible. The sample solution in the flat cell was put back into the vial immediately after measurement. The time course of the electron paramagnetic resonance (EPR) signal height of the TEMPOL signal was plotted. The EPR conditions were as follows: microwave frequency was 9.4 GHz, microwave power was 4 mW, center field was 334 mT, sweep width was 10 mT, sweep speed was 5 mT/min, modulation frequency was 100 kHz, modulation amplitude was 0.079 mT, and time constant was 0.03 s.

### Mouse thymus cell surviving

Effect of GSH-induced O_2_^•−^ to living cells was tested by a cell survival test of rodent thymus cell.^(15)^ Healthy 7-week-old Female C3H/He/Slc mice were supplied by Japan SLC, Inc. (Shizuoka, Japan). Animals were housed five per cage in climate controlled (23 ± 1°C and 55 ± 5% humidity), circadian rhythm-adjusted (12 h light–dark cycle) rooms and were allowed food and water *ad libitum* until the experiments. The mice were used for experimentation at an age of 21 weeks old (25.2 ± 2.0 g, *n* = 10). The thymus was surgically removed from a mouse. Thymocytes were squeezed out of the thymus with tweezers, put into a phosphate buffered saline (PBS), and passed through mesh to disassemble to the single cell. The cells at a density of 5 × 10^5^ cells/tube were preliminary administrated with a 1.0 mM GSH and/or 1,000 U/ml CAT and incubated at 37°C or 44°C for 2 h, and then, the cells were spin down at 600 × *g* for 5 min, re-suspended in PBS alone, and were incubated at 37°C for another 2 h. After the incubation, measurement of cell size was performed using a flow cytometry FACSCalibur (Becton, Dickinson and Company, Franklin Lakes, NJ). Experiments were carried out in compliance with and approved by the Animal Use Committee of the National Institute of Radiological Sciences, Chiba, Japan.

## Results and Discussion

**Fig. 1:**
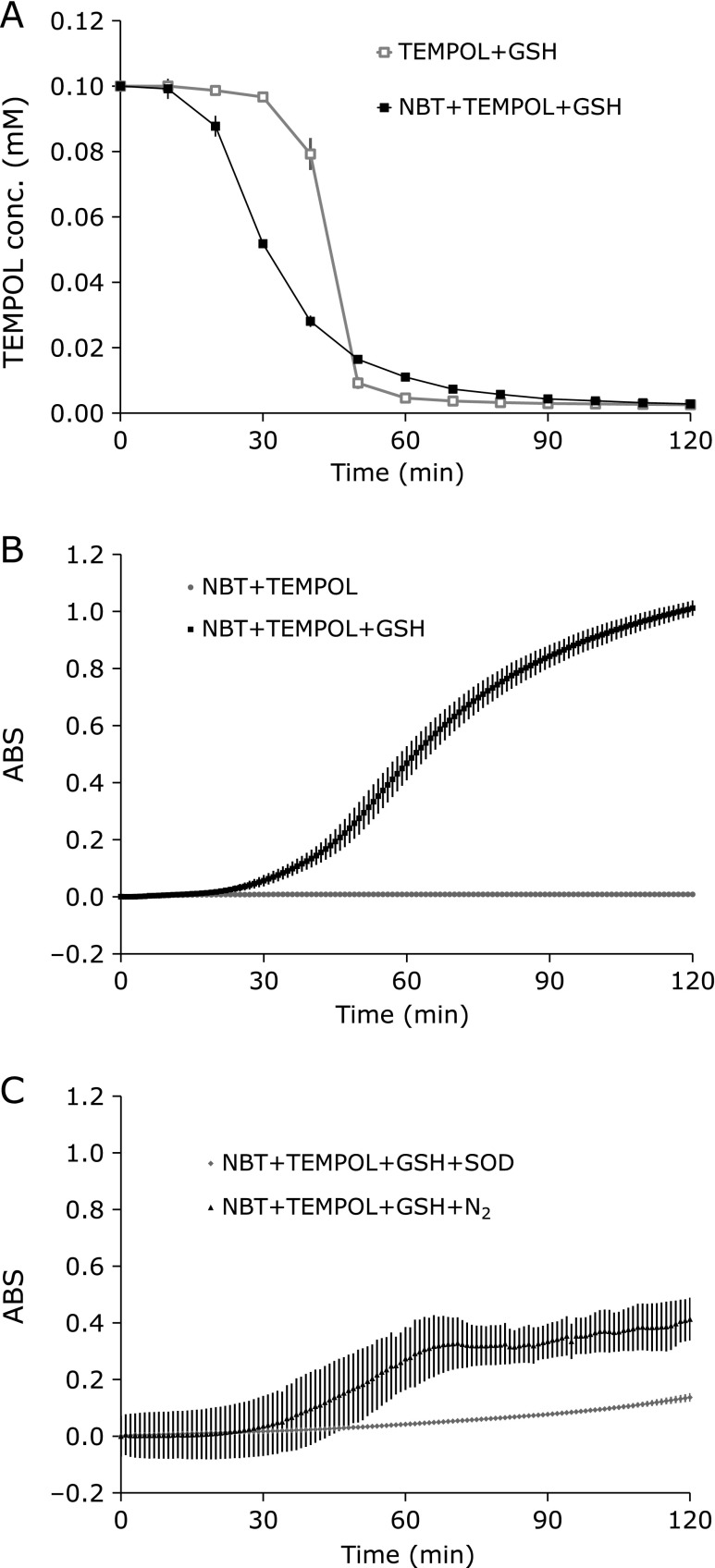
Comparison of the reaction profiles of GSH-dependent reduction of TEMPOL and the GSH-dependent formation of blue formazan (reduced NBT) at hyperthermal temperatures. (A) Reaction mixture containing 0.1 mM TEMPOL and 1.0 mM GSH was incubated at 44°C for 120 min with (black square) or without (gray square) 1.0 mM NBT. (B) Reaction mixtures containing 1 mM NBT and 0.1 mM TEMPOL were incubated with (black squares) or without 1 mM GSH (gray circles). (C) Reaction mixtures containing 1 mM NBT, 0.1 TEMPOL, and 1 mM GSH were incubated with 1.6 U/ml SOD (gray diamonds) or with bubbling N_2_ gas (black triangles). Values are indicated as averages of triplicated experiments ± SD.

For comparison with the previous paper,^(14)^ which used a basic reaction mixture formula containing 0.1 mM TEMPOL and 1 mM GSH, at first in this paper, the same formula of reaction mixture except containing 1.0 mM NBT was tested. Incubating the reaction mixture containing TEMPOL, GSH, and NBT at 44°C, the EPR signal of TEMPOL in the reaction mixture was reduced with the characteristic curve shape (Fig. 1A) similar to as shown in the previous paper.^(14)^ Simultaneously, the yellow/orange color of reaction mixture was changed to blue, and absorption at 560 nm increased, however, no increase of absorbance at 560 nm was observed without GSH (Fig. 1B). The increasing of the absorbance at 560 nm was suppressed when the 1.6 U/ml SOD was added in the reaction mixture or when the reaction mixture was bubbled by 0.5 L/min N_2_ gas (Fig. 1C). Therefore, the GSH-dependent increasing absorbance at 560 nm at 44°C in this experiment is suggesting generation of O_2_^•−^.

**Fig. 2:**
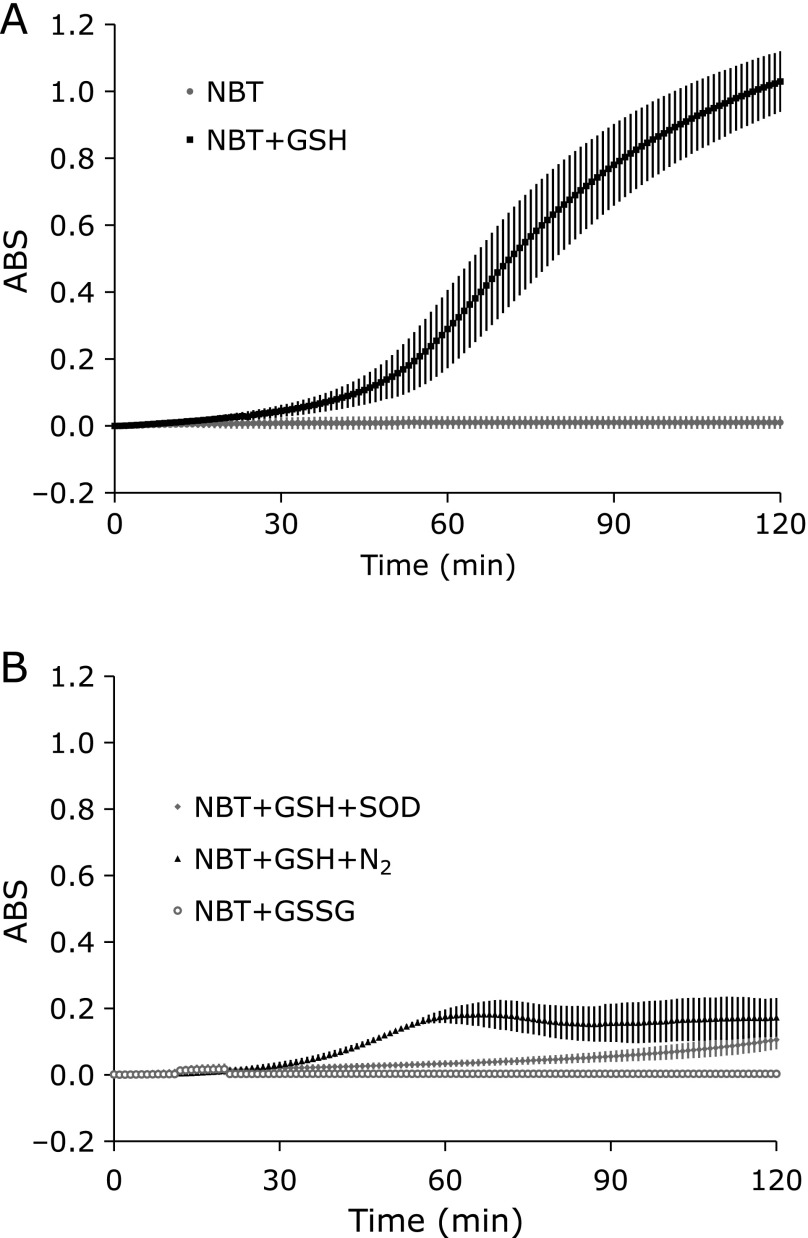
Evaluation of the effect of TEMPOL on the formation of the blue formazan (reduced NBT). The reaction mixture was incubated at 44°C for 120 min. (A) Reaction mixtures containing 1 mM NBT were incubated with (black squares) or without 1 mM GSH (gray circles). (B) Reaction mixtures containing 1 mM NBT and 1 mM GSH was incubated with 1.6 U/ml SOD (gray diamonds), with bubbling N_2_ gas (black triangles), or the case that 1 mM GSSG was added instead of GSH. Values are indicated as averages of triplicated experiments ± SD.

The source of O_2_^•−^ must be dissolved molecular oxygen (O_2_), since N_2_ bubbling in the reaction mixture could suppress the reaction (Fig. 1C and 2B). The GSH may give a hydrogen radical (^•^H), and the dissolved O_2_ could be directly reduced by a ^•^H as below to give a hydroperoxyl radical (HO_2_^•^) [Eq. 1 and 2]. The HO_2_^•^ is in equilibrium with the O_2_^•−^ in aqueous solution [Eq. 3]. Intermediating ^•^H and GS^•^ is in progress. The reactions 1, 2 and 4 may be one-step reaction. The point is that GSH can reduce O_2_ temperature dependently.

GSH ⟷ ^•^H + GS^•^  [1]

^•^H + O_2_ → HO_2_^•^  [2]

HO_2_^•^ ⟷ H^+^ + O_2_^•−^  [3]

Consequently GSSG can result from a reaction between a pair of GS^•^.

GS^•^ + GS^•^ → GSSG  [4]

The active products in this reaction are actually both HO_2_^•^, which is a relatively strong oxidant, and O_2_^•−^, which is basically a reductant.

Halliwell^(16)^ proposed a different reaction mechanism of GSH-dependent O_2_^•−^ generation, which is the reduction of O_2_ by GSSG^•−^. In their reaction, GS^•^ was initially given by a reaction of GSH and other free radical species such as ^•^OH, and then GSSG^•−^ was given by a reaction of GS^•^ and GSH. In our reaction system, however, it is quite unlikely that the other free radicals participate the formation of GS^•^ and/or ^•^H.

The actual trigger of the TEMPOL reduction should be one-electron-oxidation of TEMPOL by HO_2_^•^ to give an oxoamonium cation form of TEMPOL. The oxoamonium cation form of TEMPOL then could react with GS^•^ to make a stable non-paramagnetic complex and as a result TEMPOL was two-electron-reduced.^(13)^ On the other hand, NBT can be directly reduced by O_2_^•−^ to give blue formazan dye. Strong reductants, such as ascorbic acid, can also reduce NBT and give the blue formazan (data not shown).

Adding NBT in the reaction mixture made the decaying TEMPOL a little faster and the delay time shorter (Fig. 1A). The nitroxyl radical form of TEMPOL can be one-electron-oxidized by HO_2_^•^ to be the oxoamonium cation form. In an opposite way, the oxoamonium cation can be one-electron-reduced by O_2_^•−^ to recover the nitroxyl radical. The reduction of NBT by O_2_^•−^, which can also be recognized as the consumption of O_2_^•−^ by NBT, may suppress reduction of the oxoamonium cation form to a nitroxyl radical form, and apparently accelerate one-electron-oxidation of TEMPOL to the oxoamonium cation form. As a result, quasi-one-electron-reduction of TEMPOL, i.e., decay of the EPR signal of TEMPOL, could be accelerated. In this case NBT and the oxoamonium cation form of TEMPOL competes each other for O_2_^•−^. NBT may decrease the population of O_2_^•−^, but not deplete and allow the presence of HO_2_^•^. SOD, however, may deplete O_2_^•−^, then the equiribrium between HO_2_^•^ and O_2_^•−^ [Eq. 3] would strongly move to the right. Therefore, SOD does not allow that HO_2_^•^ could exist. In other words, HO_2_^•^ rapidly changed to O_2_^•−^ before reacting with other molecules, and then was deleted by SOD.

The next experiment tested whether the TEMPOL had any effect on the generation of O_2_^•−^. When the reaction mixture containing simply GSH and NBT was incubated at 44°C, the similar increasing of absorbance at 560 nm shown in Fig. 1A was observed at almost the same timing (Fig. 2A). However, no increasing absorbance at 560 nm was observed without GSH (Fig. 2A). The increasing of the absorbance at 560 nm was suppressed when the 1.6 U/ml SOD was added in the reaction mixture or when the reaction mixture was bubbled by 0.5 L/min N_2_ gas (Fig. 2B). Since reaction profiles in Fig. 1 and 2 can be evaluated almost similar shapes in considering the variability, the TEMPOL in the reaction mixture has no modification as to the generation of O_2_^•−^. This result certified that the TEMPOL was working just as a redox detector in the previous paper.^(14)^

No increase of absorbance at 560 nm was also observed when GSSG was used instead of GSH (Fig. 2B). This result is probably the same as the previous paper,^(3)^ which demonstrated that no TEMPOL reduction was observed when TEMPOL was incubated at 70°C with GSSG instead of GSH, even though the detection method was different to this paper. Oxygen can not extra-oxidize GSSG to make O_2_^•−^ even in a higher temperature. In other words, it can say that GSSG is a stable enough end product of the anti-oxidative process of GSH. In consequence of stability of the GSSG, the enzymatic system is required to recover GSH from GSSG.

Adding 1.6 U/ml SOD or 0.5 L/min N_2_ gas bubbling could stop the quasi-one-electron-reduction of TEMPOL, i.e., EPR signal decay of TEMPOL, in the previous paper. However, a slight increase of absorbance at 560 nm, reduction of NBT, was still observed in the experiment with 1.6 U/ml SOD or 0.5 L/min N_2_ gas bubbling (Fig. 1C and 2B) in this paper. In the hypoxic condition, GSH could be able to reduce NBT directly rather reduce O_2_. Since this direct reduction of NBT by GSH in hypoxic condition looks saturated, the reaction is reversible. The direct reduction of NBT by GSH in the hypoxic condition might be not negligibly slow.

**Fig. 3:**
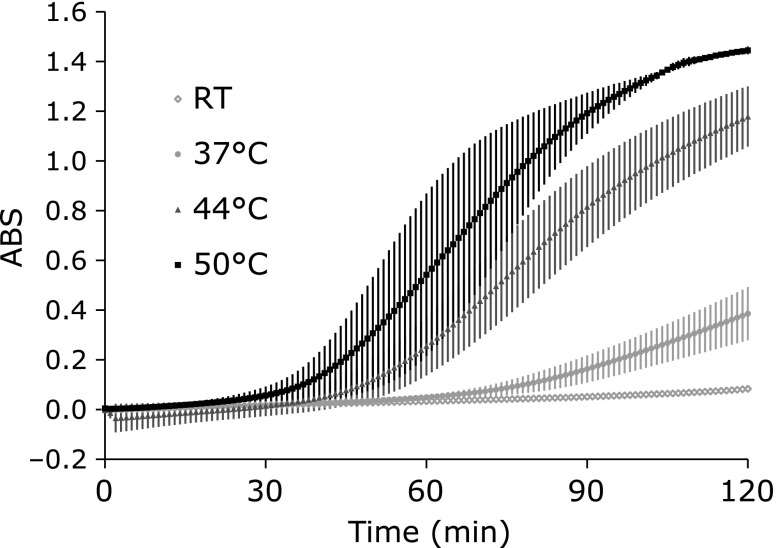
Temperature dependent formation of the blue formazan (reduced NBT). The reaction mixture containing 1.0 mM NBT and 1.0 mM GSH was incubated at 50°C, 44°C, 37°C, or room temperature for 120 min. Values are indicated as averages of triplicated experiments ± SD.

Next, the temperature dependence of the NBT-GSH reaction was tested. The GSH dependent NBT-derived blue formazan generation was exaggerated temperature dependently (Fig. 3). The results of Fig. 2 and 3 suggest that GSH has worked on the temperature dependent generation of HO_2_^•^/O_2_^•−^.

**Fig. 4:**
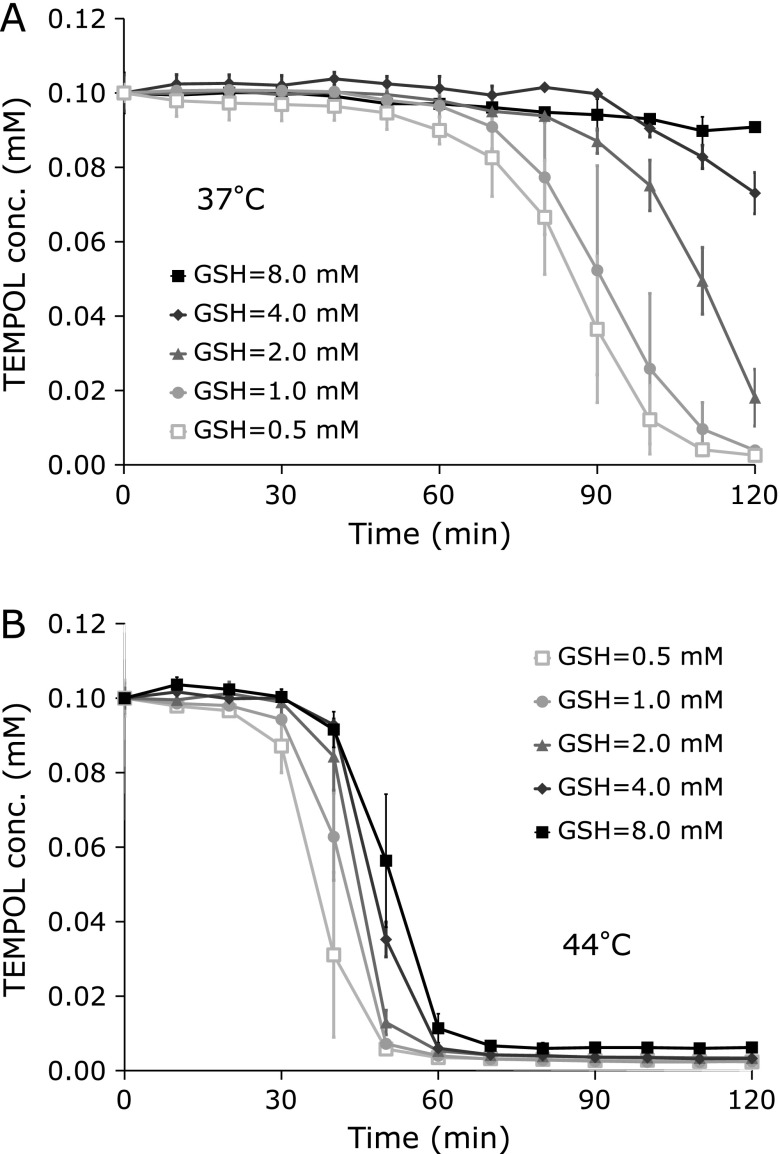
Effect of GSH concentration on the reduction of TEMPOL. The reaction mixtures containing 0.1 mM TEMPOL and several different GSH concentrations were incubated at (A) 37°C or (B) 44°C for 120 min. Values are indicated as averages of triplicated experiments ± SD.

When concentration of GSH in the reaction mixture containing 0.1 mM TEMPOL was varied from 0.5–8.0 mM, decay of TEMPOL became slower depending on the GSH concentration (Fig. 4A and B). At 37°C, TEMPOL reduction was almost stopped by 8.0 mM GSH. At 44°C, the effect of GSH concentration was mild compared to the experiment at 37°C. The timing of steep decay of TEMPOL reduction was delayed when GSH concentration increased. GSH itself may able to reduce HO_2_^•^ directly, in other words the HO_2_^•^, a relatively strong oxidant, can oxidize GSH as [Eq. 5].

2GSH + 2HO_2_^•^ → GSSG + 2H_2_O_2_  [5]

In short, GSH can consume HO_2_^•^, and suppress the process of one-electron-oxidation of TEMPOL. GSH and TEMPOL competes with each other for HO_2_^•^.

In the previous paper,^(14)^ the steep decay of TEMPOL following the initial delay time was shift to the right, i.e., delayed, by adding albumin and/or ascorbic acid. Albumin, which can be readily oxidized has been known as an antioxidative biomolecule. Ascorbic acid has been known as relatively strong reductant, which can be easily oxidized in other words. Both molecules, therfore, can be oxidized by HO_2_^•^. This can be said that the both molecules can consume HO_2_^•^. Initial consumption of HO_2_^•^ can delay the reaction and push the slope to the right.

**Fig. 5:**
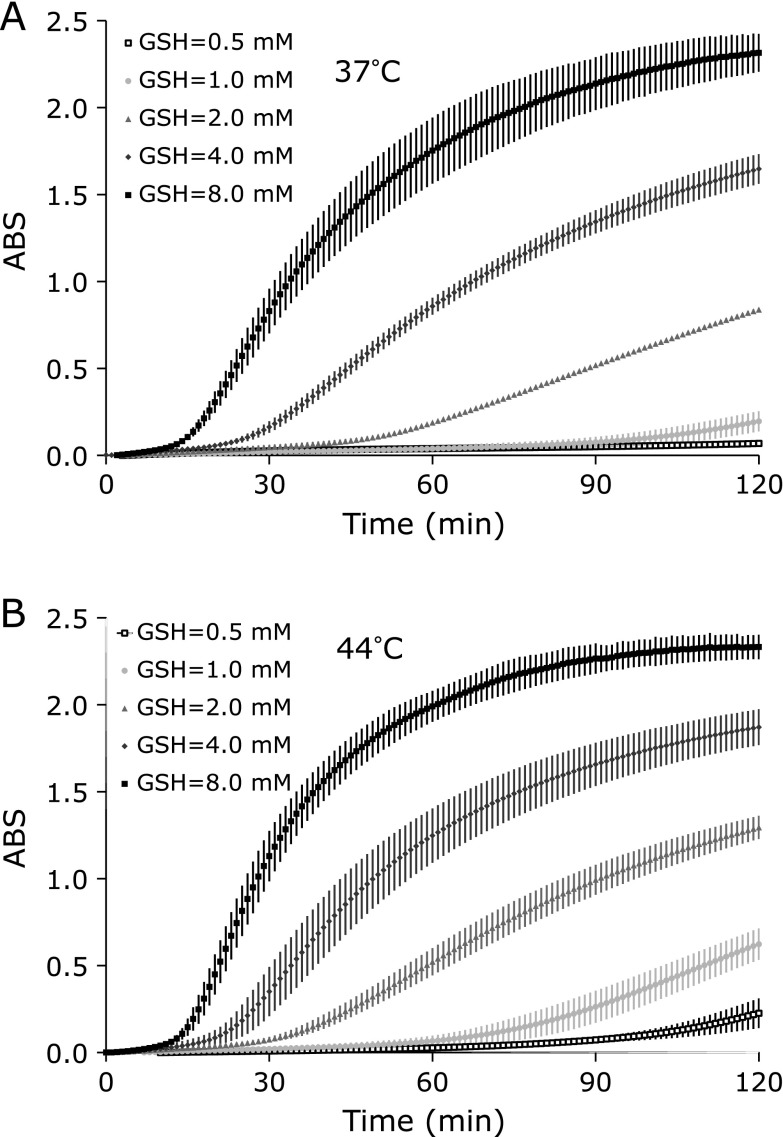
Effect of GSH concentration on the formation of the blue formazan (reduced NBT). The reaction mixtures containing 1 mM NBT and several different GSH concentrations were incubated at (A) 37°C or (B) 44°C for 120 min. Values are indicated as averages of triplicated experiments ± SD.

In an interesting twist, the formation of the blue formazan, i.e., absorbance at 560 nm, increased when the GSH concentration increased in the reaction mixture containing NBT (Fig. 5). The maximum level of blue formazan formation with 8 mM GSH at 120 min incubation was similar level in either 37°C or 44°C experiment, although the reaction at 44°C was slightly faster than that at 37°C. However, the maximum level of blue formazan formation with 0.5 or 1.0 mM GSH at 120 min incubation was around 3 times more at 37°C compared to 44°C experiment. In addition, the formation of blue formazan with 8 mM GSH was suppressed only around 10% by adding 3.2 U/ml SOD in the reaction mixture. This is more like direct reduction of NBT by GSH.

Reaction of NBT and O_2_^•−^ may be sufficiently faster than consumption of HO_2_^•^ by GSH. NBT can consume O_2_^•−^ and brake an equivalent balance of the HO_2_^•^/O_2_^•−^ population. Therefore, HO_2_^•^ must be rapidly changed to O_2_^•−^ before being reduced by GSH. The reduction of NBT can be exaggerated by increasing GSH concentration, which can work not only direct reduction of NBT but also work on increasing O_2_^•−^ production.

Additionally to the [Eq. 5], H_2_O_2_ could be yielded by the reaction of a pair of HO_2_^•^ [Eq. 6], and then the H_2_O_2_ could consume the O_2_^•−^.

HO_2_^•^ + HO_2_^•^ → H_2_O_2_ + O_2_ [6]

H_2_O_2_ + O_2_^•−^ → OH^−^ + ^•^OH + O_2_ [7]

The reaction of [Eq. 7] can make a hydroxyl radical (^•^OH). This reaction may be harmful due to the high reactivity of the product, i.e., ^•^OH, if the reaction occurs in an intracellular space. However, the extracellular generation of ^•^OH may not be lethally important, since ^•^OH could immediately be deleted by extracellular molecules due to its super-high reactivity and could not reach to the intracellular bio-functional molecules. Membrane permeable HO_2_^•^ and/or H_2_O_2_ can have a chance to give lethal effects on cells.

To confirm the reaction of [Eq. 5, 6, and 7], the effect of CAT and H_2_O_2_ was tested. Addition of CAT to the reaction mixture enhanced the maximum amount of the blue formazan formation dose dependently (Fig. 5A). Conversely, addition of H_2_O_2_ to the reaction mixture suppressed the formation of the blue formazan with dose dependent manner (Fig. 5B). These results suggest that the generation of H_2_O_2_ in the reaction mixture was expected and then the H_2_O_2_ is consuming the GSH-induced O_2_^•−^. The trigger of the GSH-dependent TEMPOL reduction is one-electron-oxidation of TEMPOL by HO_2_^•^. Catalase could slightly accelerate GSH-dependent TEMPOL reduction but not significantly (data not shown), since elimination of H_2_O_2_, which is a consumer of O_2_^•−^, can move the population of [Eq. 3] left.[Fig F6]

**Fig. 7:**
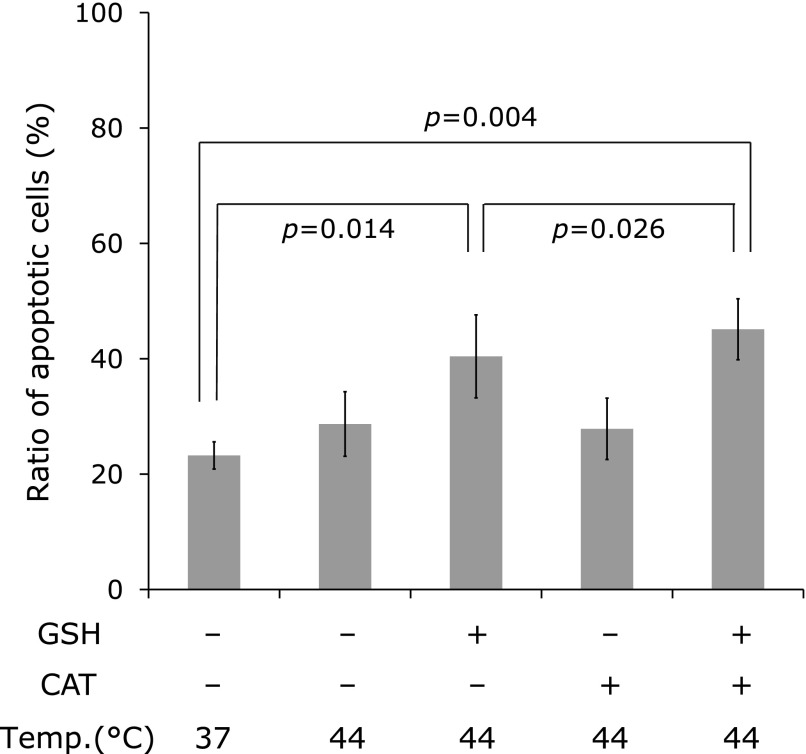
Effect of coexisting GSH and catalase with a hyperthermic stress task to the mouse thymus cell culture. Mouse thymus cells were incubated with 1 mM GSH at 44°C for 120 min with or without catalase. Then the cells were washed and again incubated at 37°C for an extra 120 min. Values are indicated as averages of triplicated experiments ± SD. Significance was certified when the calculated *p* value was less than 0.05.

Fig. 7 shows the result of the cell survival test using mouse thymus cells. Incubating thymus cells at 44°C for 2 h in PBS could cause apoptosis, i.e., cell size shrank. Incubating thymus cells at 44°C for 2 h in PBS containing 1 mM GSH caused higher apoptosis levels. Incubating thymus cells at 44°C for 2 h in PBS containing 1 mM GSH and 1,000 U/ml catalase caused apoptosis more and the percentage of shrunk cells increased.

**Fig. 8:**
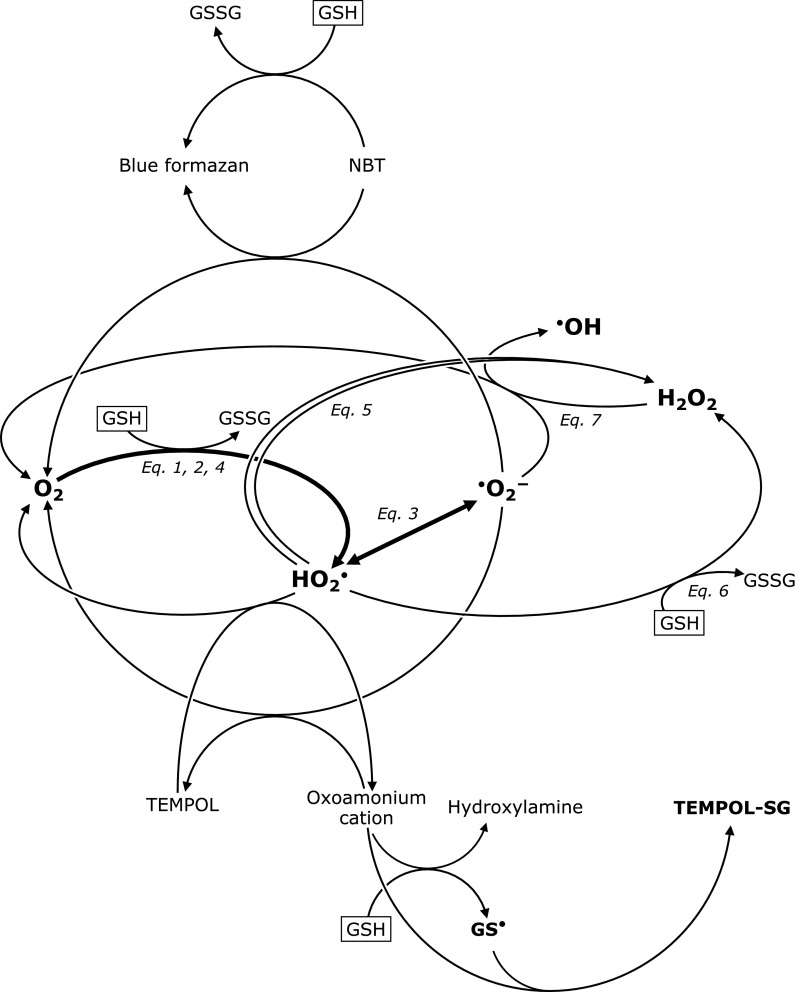
A schematic drawing of reactions observed in this paper.

Fig. 8 shows a schematic drawing of reactions observed in this paper. GSH may reduce O_2_ to make HO_2_^•^, which is equilibrated with O_2_^•−^ in an aqueous condition. TEMPOL or NBT was used as a detector of GSH-induced HO_2_^•^ or O_2_^•−^ generation at hyperthermal temperatures. However, GSH itself can affect the estimation of GSH-induced HO_2_^•^ or O_2_^•−^, by reacting with NBT or HO_2_^•^. HO_2_^•^ can be a source of H_2_O_2_, which can consume O_2_^•−^. The reactions were actually quite complicated despite the limited number of players; due to the GSH wearing more than one hat. After all, GSH is still a difficult compound which interferes with detecting self-created HO_2_^•^/O_2_^•−^.

GSH exists in living tissues in mM level. The *in vivo* oxygen level can cause GSH-induced TEMPOL reduction,^(14)^ i.e., GSH-induced HO_2_^•^ or O_2_^•−^ generation, at hyperthermal temperatures. This reaction may be a key for hyperthermia effects. The rapid proceeding of the reaction shown in this *in vitro* experiment may not go in the living cells, since HO_2_^•^/O_2_^•−^ can not accumulate in living cells. However GSH-induced HO_2_^•^/O_2_^•−^ may constantly occurred in *in vivo* situations and it can be accelerated temperature dependently.

The result in this paper suggests that coexisting catalase with GSH in a reaction mixture could exaggerate GSH-induced O_2_^•−^ generation at hyperthermal temperatures. Exaggerated extracellular O_2_^•−^ generation could give a harmful effect to living cells.

## Conclusion

Temperature dependent generation of O_2_^•−^ in an aqueous sample containing GSH was again confirmed by the NBT method. GSH may reduce O_2_ to make HO_2_^•^, which is equilibrated with O_2_^•−^ in an aqueous condition. H_2_O_2_ was also generated simultaneously in this reaction mixture from HO_2_^•^, and then H_2_O_2_ consumed O_2_^•−^. Coexisting catalase in the reaction mixture, as a result, could increase O_2_^•−^ generation. The catalase-exaggerated extracellular O_2_^•−^ generation could give a harmful effect to living cells. This GSH-induced oxidative stress can be a part of mechanisms of hyperthermia therapy.

## Figures and Tables

**Fig. 6 F6:**
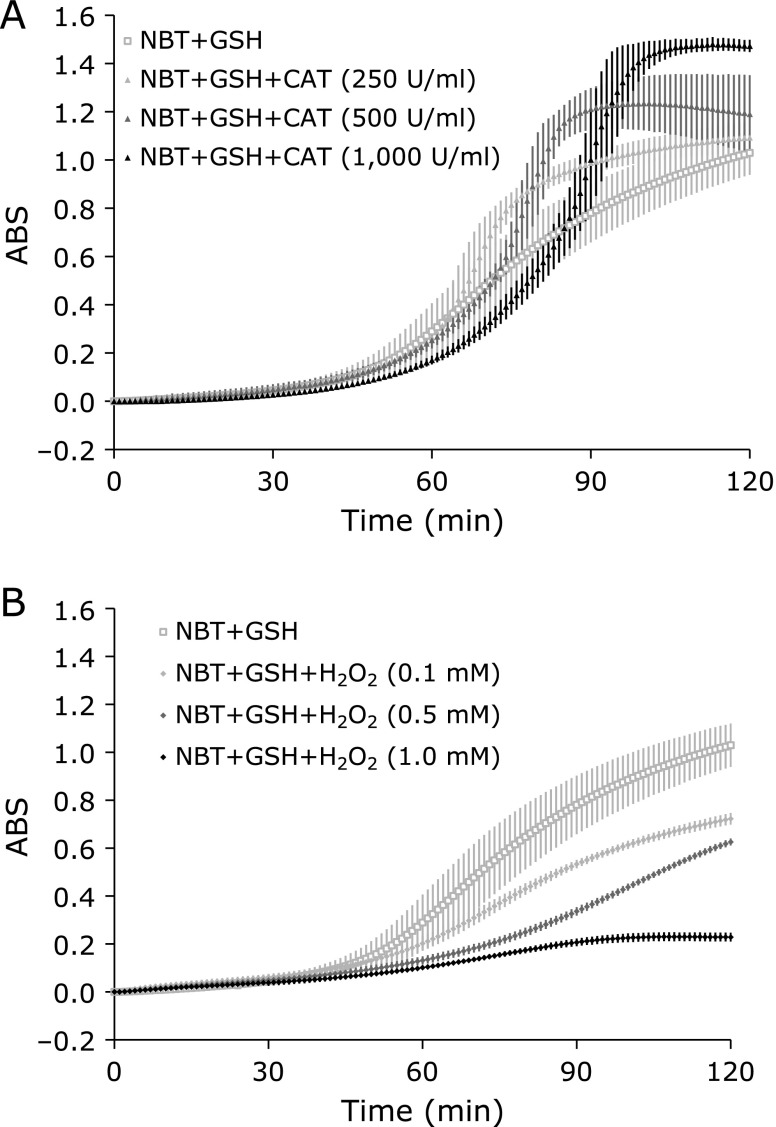
Effect of catalase and H_2_O_2_ on the formation of the blue formazan (reduced NBT). (A) Effect of catalase. The reaction mixture containing 1.0 mM NBT and 1.0 mM GSH was incubated at 44°C for 120 min with several concentrations of catalase. (B) Effect of H_2_O_2_. The reaction mixture containing 1.0 mM NBT and 1.0 mM GSH was incubated at 44°C for 120 min with several concentrations of H_2_O_2_. Values are indicated as averages of triplicated experiments ± SD.

**Table 1:** Composition of reaction mixtures tested

No.	NBT (mM)	TEMPOL (mM)	GSH (mM)	Other conditions	Result
1	—	0.1	1.0	—	Fig. 1A
2	1.0	0.1	1.0	—	Fig. 1A
3	1.0	0.1	—	—	Fig. 1B
4	1.0	0.1	1.0	—	Fig. 1B
5	1.0	0.1	1.0	1.6 U/ml SOD	Fig. 1C
6	1.0	0.1	1.0	0.5 L/min N_2_ bubbling	Fig. 1C

7	1.0	—	—	—	Fig. 2A
8	1.0	—	1.0	—	Fig. 2A
9	1.0	—	1.0	1.6 U/ml SOD	Fig. 2B
10	1.0	—	1.0	0.5 L/min N_2_ bubbling	Fig. 2B
11	1.0	—	—	1 mM GSSG	Fig. 2B

12	1.0	—	1.0	at 50°C, 44°C, 37°C, or RT	Fig. 3

13	—	0.1	0.5–8.0	at 37°C	Fig. 4A
14	—	0.1	0.5–8.0	at 44°C	Fig. 4B

15	1.0	—	0.5–8.0	at 37°C	Fig. 5A
16	1.0	—	0.5–8.0	at 44°C	Fig. 5B

17	1.0	—	1.0	250 U/ml Catalase	Fig. 6A
18	1.0	—	1.0	500 U/ml Catalase	Fig. 6A
19	1.0	—	1.0	1,000 U/ml Catalase	Fig. 6A
20	1.0	—	1.0	0.1 mM H_2_O_2_	Fig. 6B
21	1.0	—	1.0	0.5 mM H_2_O_2_	Fig. 6B
22	1.0	—	1.0	1.0 mM H_2_O_2_	Fig. 6B

RT, room temperature.
